# Supplementary material for: Are carbon emissions trading and green financial instruments synergistic? -Comprehensive quantitative research based on content analysis
Source: PLoS One. 2024 Mar 7;19(3):e0298601. doi: 10.1371/journal.pone.0298601 (PMC10919601; doi:10.1371/journal.pone.0298601)
Supplement: S1 Data — (ZIP) [file pone.0298601.s001.zip › Data/地级市Z_F工作报告.zip!/╕ú╜¿/╒─╓▌╩╨/└┤╫╘╣1⁄2╓┌║┼í╛┬φ┐╦╩2╛▌═°í┐_2009.docx]

各位代表：

现在，我代表漳州市人民政府向大会作政府工作报告，请予审议，并请市政协委员和其他列席人员提出意见。

　　一、全面完成2008年各项目标任务

　　2008年，在省委、省政府和市委的正确领导下，在市人大法律监督、工作监督和市政协民主监督下，我市各级政府认真贯彻党的十七大、十七届三中全会精神，以邓小平理论和“三个代表”重要思想为指导，以科学发展观为统领，以解放思想为先导，以改革开放为动力，围绕全省建设两个先行区的目标要求，突出“海西建设、漳州先行”发展主线，深入实施“依港立市、工业强市、开放活市、科教兴市”发展战略，努力克服国内外经济形势复杂多变带来的冲击，全市经济社会发展呈现增长较快、结构优化、后劲增强、民生改善的良好态势，全面完成市十四届人大三次会议确定的各项目标任务。

——综合实力跃上新的台阶。针对经济发展中面临的困难，相继出台扶持中小企业经营发展十六条、减轻企业负担二十条、稳定房地产市场十条和扩大内需十个方面等政策措施，促进经济持续较快增长，“两个突破”目标顺利实现，综合实力跃上新的台阶。地区生产总值1002.01亿元，增长13.6%；财政总收入101.58亿元，增长21.2%，其中地方级财政收入60.49亿元，增长27.6%；农业总产值380.42亿元，增长5.6%；工业总产值1451.14亿元，增长20.1%，其中规模工业产值1245.14亿元，增长22.5%；全社会固定资产投资438.65亿元，增长34.1%；实际利用外资5.01亿美元，增长11.2%；外贸出口38.76亿美元，增长13.4%；本外币各项存款余额719.62亿元，增长17.2%，各项贷款余额509亿元，增长15.6%。地区生产总值、人均地区生产总值、地方级财政收入、新型农村合作医疗参合率和城镇职工基本养老保险覆盖率提前两年完成“十一五”规划目标。

——经济运行质量持续提升。三次产业比例由21.8∶43.2∶35.0调整为21.3∶44.5∶34.2。财政总收入占GDP比重为10.14%，提高0.33个百分点。工业经济效益综合指数170%，提高13个百分点。科技创新加快推进，荣获全国科技进步考核先进市，11个县（市、区）再次全部通过全国科技进步考核，万利达入选全国首批创新型企业，全市高新技术企业实现产值255.87亿元，增长9.4%。工业“千百工程”建设超额完成，产业集群加速形成，全市“3+4”产业实现产值820.29亿元，占全市规模工业产值的65.9%，增长22.1%，其中食品、汽车、船舶工业产值分别增长40.5%、18.0%、74.2%。民营经济发展强劲，民营规模工业实现产值458.17亿元，增长36.0%，民营投资177.15亿元，增长41.4%。品牌带动效应凸显，新增中国驰名商标7个，全市24家拥有中国名牌和驰名商标规模工业企业产值161.39亿元，增长31.4%。三产带动能力增强，实现社会消费品零售总额363.78亿元，增长20.3%。旅游业加快发展，新增云洞岩、龙佳生态温泉山庄2个国家4A级景区，东山县被评为优秀旅游县。

　　——港口经济发展取得突破。招银港区7号泊位、古雷一德石化码头、力通5000吨级滚装码头、华福3000吨级码头投入生产，海洋石化25万吨级码头、古雷10万吨级航道（一期）、东山港区大东3000吨级化工码头、福建能源5000吨级化工码头、旗滨公共码头等一批配套临港工业项目抓紧施工，后石港区30万吨级大型泊位、福欣特殊钢2个3万吨级泊位正在加快推进，厦漳跨海大桥、厦漳高速公路扩建工程漳州段开工建设，324国道80公里路面完成改造。《古雷区域发展建设规划》获省政府批准，福欣特殊钢建设有序推进，后石电厂七号机组并网发电，金龙客车整车项目建成投产，旗滨玻璃第一条生产线点火生产，台湾玻璃、长春二期、凯景钢铁扩建、湘电风能等一批临港工业项目加快建设。港口物流业快速发展，全市港口货物吞吐量3410万吨，集装箱吞吐量39.2万标箱，分别增长20.4%、46.5%。

　　——现代农业发展步伐加快。农业产业化加快发展，全市现有国家级重点龙头企业4家，省级龙头企业53家，市级龙头企业141家，农产品加工营销企业2895家，农业专业户2.3万户。“六千”水利工程超额完成，防洪排涝河港清淤整治完成165公里，海洋与渔业减灾防灾“百千万”工程有效推进，防灾减灾体系进一步健全。水仙花获中国驰名商标称号，民光牌蜜柚获中国名牌农产品称号，4家企业获“福建省品牌农业企业金奖”，新增28个无公害农产品、20个绿色食品、3个有机食品，6个产品获原产地标记注册保护，2个产品获国家地理标志保护，云霄被评为中国优质枇杷基地重点县。漳荷花卉产业合作进展良好，闽荷花卉交流合作中心加快建设，闽荷花卉合作（荷兰）有限公司暨漳州市花卉产业办驻荷兰代表处正式挂牌，花卉出口2062.2万美元，增长16.4%。

　　——漳台交流合作全面拓展。实际利用台资2.43亿美元，占验资总额的48.6%，增长23.4%。组织34批次人员登岛招商，加强两地产业对接和文化交流。成功举办第十届花博会、台湾农产品博览会、第二届中国蘑菇节、漳台经贸恳谈会、开漳圣王文化节、关帝文化旅游节、保生大帝文化节、平和琯溪蜜柚节、诏安青梅节、首届福建土楼（南靖）文化节等一系列节庆活动，第十届花博会共签约外资项目59个、合同金额2.45亿美元，创下历届之最。海峡两岸（福建东山）水产品加工集散基地建设加快推进，台商综合服务大楼规划建设，南太武滨海工业集中区规划加快编制。

——各项改革继续纵深推进。农村综合改革进一步深化，土地使用权流转进程加快，全市共流转土地10.05万亩，集体林权确权发证工作深入开展，海域使用权市场化配置稳步推进，水利工程管理体制改革加快推进。工业安装公司、龙溪内拖配件厂等企业完成改制重组，漳龙公司发行10亿元企业债券正式上报国家发改委核准，闽商（漳州）创业投资有限公司正式成立。财政管理体制改革深入推进，国库集中支付改革全面推开。政府职能加快转变，新行政服务中心有效运转，公共资源交易中心有序运行。

　　——城乡面貌发生明显变化。创建国家卫生城市正式获得授牌，国家园林城市顺利通过复核检查。组织编制《漳州中心城市拓展概念规划》，中心市区规划建成区面积从100平方公里扩大到300平方公里，推动漳州从滨江城市向滨海城市发展。全市投入22.38亿元用于城市基础设施建设，其中市区投入13.95亿元，建成漳华路、江滨路西延伸段等八条道路，道路网络体系明显改善，交通拥堵现象得到缓解。疏浚九十九湾及其支流46公里，新辟公交线路3条，增加公交车70辆。新建农村水泥路480公里，完成43座危桥改造。43个新农村建设示范村加快建设，完成“种植名贵树木、建设绿色家园”示范村建设41个，建设绿色通道298公里，植树造林28万亩。农村“家园清洁行动”和“家园整理行动”深入开展，24个镇135个村通过省级验收，农村环境卫生明显改观。纳入规划124个村级组织活动场所全部建成。1个县、4个乡镇通过国家级生态示范县、国家级环境优美乡镇验收命名。

　　——节能减排工作成效显著。深入贯彻节能法，完善差别电价、替代发电等措施，重点节能工程建设加快推进。全市列入省重点减排项目的5座污水处理厂建成，配套铺设污水管网130公里，市区生活污水集中处理率达73.46%。医疗废弃物处置中心、建筑垃圾处置场建成投入使用。编制饮用水源保护规划，加大饮用水源环境整治力度，完成7个县级水源保护区围网建设以及38个乡镇水源保护区界标、道路警示牌设置。深入开展打击违法排污行为、保障人民群众健康专项行动，完成7家挂牌督办企业污染治理任务，依法关闭高污染、高耗能、安全生产不达标的企业22家，对42家省市重点污染企业安装在线监控设施。清洁生产加快推进，云陵开发区列入省循环经济示范园区。九龙江和海洋环境污染整治力度加大，九龙江漳州段水质达标率95.8%。全年万元GDP能耗下降2.6%，主要污染物排放量控制在省政府下达指标之内。

　　——人民群众生活继续改善。物价指数得到有效控制，全年上涨4.6%。城镇居民人均可支配收入16023元，实际增长10.8%；农民人均纯收入6506元，实际增长9.2%。35个为民办实事项目超额完成年度投资计划，城乡低保对象实现应保尽保，残疾人等弱势群体基本生活得到妥善安置。新增城镇职工养老保险4.86万人、医疗保险5.44万人。城镇居民医疗保险全面铺开，参保人数44.34万人。农村新型合作医疗参合农民333.97万人，参合率92.3%，补助标准提高到80元，报销手续更加便捷。新增就业5.43万人，下岗失业人员实现再就业1.25万人，转移农村劳动力7.75万人，城镇登记失业率3.08%。新建经济适用房2603套、廉租住房52套，发放廉租房租金318.73万元，新增廉租住房保障1345户。投入1.11亿元，完成“侨居造福工程”2119套住房建设任务。农房保险、被征地农民养老保险、农民工工伤保险、渔工政策性保险、渔船保险、农村自然灾害公众责任保险等工作顺利推进。

　　——社会各项事业加快发展。土楼申报世界文化遗产获得成功，闽南文化生态保护实验区建设扎实推进，第三次文物普查进展顺利。《全民科学素质行动计划纲要》深入实施。义务教育“两免一补”政策落到实处，受惠学生50多万人。“双高普九”有序推进，中小学布局调整优化，筹建漳州实验小学新校区，市区可新增小学生容量4000人以上。投入4312万元完成中小学危改项目47个，投入3949万元建成农村寄宿制学生宿舍、食堂共70个项目。高考本科上线13261人，增长26.15%。投入4000多万元改造提升39所重点乡镇卫生院和16所社区卫生服务中心。全民健身与奥运同行系列活动蓬勃开展，四名运动员获选参加北京奥运会、残奥会并取得好成绩，市运会筹备工作有序推进。低生育水平保持稳定，出生人口政策符合率明显提高，人口自然增长率6.74％。。强化国土资源管理，严格执行耕地保护政策，继续实现耕地占补平衡。妇女儿童、老干老龄、老区建设、红十字会、民族宗教、外事侨务、新闻出版、广播电视、统计、物价、史志、档案、地震、人防、气象、残联、国防动员、支前双拥等各项工作都取得新成效。

　　——民主法治建设得到加强。以“爱我漳州、从我做起”宣传教育为主线，深入开展社会主义荣辱观教育，深化文明城市（县城、城区）、文明行业和文明和谐示范村等创建活动，树立一批精神文明建设先进典型，长泰荣获首批“全国文明县城”，不断激发广大市民建设漳州的热情。主动向市人大及其常委会报告工作，向市政协通报情况，办理市人大代表建议115件、市政协提案278件，办结率均为100%。广泛听取各民主党派、工商联、无党派人士及社会各界人士意见。深入贯彻《全面推进依法行政实施纲要》，行政执法责任制全面推行，相对集中行政处罚权工作成效明显，行政决策程序更加健全。“五五”普法活动深入开展，行政复议工作得到加强，政府信息公开制度积极推进，政府门户网站逐步完善，政务公开范围不断拓展。深化机关效能建设，细化“重在提请、主动服务”，出台一系列便民利企新举措，政府服务发展、服务群众的质量进一步提升。加强政府系统反腐倡廉建设，强化行政监察、审计专项监督，加大预防和惩治腐败体系建设力度，全面规范行政自由裁量权，积极探索规范行政权力运行工作，清理整顿、规范发展中介组织，推进公共资源市场化配置，坚决纠正损害群众利益的不正之风，政风行风有新好转。

　　——社会形势保持和谐稳定。综治责任制有效落实，“平安漳州”建设成效明显，公众安全感满意率94.15%，综治考核名列全省第二名。集中开展重信重访、非正常上访等信访突出问题专项治理，加强矛盾纠纷排查调处，完善信访工作机制，有效预防和处置各类群体性事件，确保北京奥运会等重大活动期间的社会稳定。健全应急预案和应急救助体系，应对突发事件能力不断增强。加强“餐桌污染”治理和“食品放心工程”建设，开展食品药品质量安全专项整治，认真抓好乳制品质量安全专项清查，全力做好患病婴幼儿筛选救治工作。狠抓安全生产专项整治，强化安全隐患源头治理，安全生产四项控制指标全面下降。

　　——支援地震灾区有力有效。大力发扬“一方有难、八方支援”的精神，全市累计捐款捐物8883.67万元。派出55名医疗防疫人员赴灾区执行任务，圆满完成79名灾区伤病员救治任务。按照省里统一部署，义不容辞承担起对口支援彭州市敖平、三界两镇恢复重建任务。组织4000多名建筑工人赴彭州市参与灾后重建，提前完成过渡安置房4182套8.77万平方米安装任务。已拨付援建资金7150万元，援建项目漳州水仙路、九龙江路、龙江路和三界镇公立卫生院已竣工，敖平中学已动工建设。邀请彭州市参加第十届花博会，免费提供展位，帮助开展招商，支持灾区加快重建步伐。

　　各位代表，过去的一年，我市在国际经济环境迅速恶化、国内经济困难明显加大的情况下，战胜了比预想要大的困难，取得了比预期要好的成绩，这是市委正确领导、全市人民共同努力、方方面面大力支持的结果。在此，我代表市人民政府向辛勤工作在各行各业的广大干部群众，向给予政府工作大力支持的人大代表、政协委员、各民主党派、工商联、离退休老同志和社会各界人士，向积极服务漳州发展的中央、省驻漳单位和驻漳部队、武警官兵，向为漳州发展做出突出贡献的企业家、投资者和建设者，向关心支持漳州发展的港澳台同胞、海外侨胞和国际友人，表示衷心的感谢并致以崇高的敬意！

　　在看到成绩和进步的同时，我们也清醒地认识到我市经济社会发展还存在不少困难和问题。主要有：受国际金融危机影响，部分经济指标增速放缓，企业出口受到严重冲击，一些行业和中小企业生产经营困难，保持经济增长压力明显加大；经济综合实力仍然较弱，现代产业体系尚不完善，经济结构不尽合理，自主创新能力不强，新兴服务业发展相对滞后；农业支持保护体系不够健全，农村基础设施较为薄弱，农资价格上涨过快，农业增效、农民增收难度加大；财政收支矛盾突出，改善公共服务的任务仍然繁重，与群众切身利益密切相关的就业、教育、卫生、文化、社会保障等领域投入依然不足；安全生产仍有隐患，食品药品质量安全存在薄弱环节，节能降耗、环境保护任务仍然艰巨；政府职能转变尚未完全到位，社会管理和公共服务能力有待增强，部分公务人员工作效率不高、作风不够扎实，官僚主义、形式主义、奢侈浪费、腐败现象在一些地方和部门仍然比较突出等等。对于这些困难和问题，我们一定高度重视并认真加以解决。

　　二、全力保持2009年经济社会持续较快发展

　　今年是新中国成立60周年，是深入学习实践科学发展观、推进漳州先行的重要一年。面对复杂的形势、严峻的挑战，我们要把保持经济持续较快发展作为首要任务，主动从挑战中捕捉发展机遇、从逆境中培育有利因素、从应变中拓展作为空间，着力在扩大内需、城乡一体、产业培育、改革开放、生态建设、改善民生等方面求突破，全力推进漳州又好又快发展。

　　今年政府工作的总体要求是：坚持以邓小平理论和“三个代表”重要思想为指导，全面贯彻党的十七大、十七届三中全会和中央、省经济工作会的精神，深入落实科学发展观，围绕推进海峡西岸经济区“两个先行区”建设，按照省委“四求先行”要求，继续坚持“海西建设、漳州先行”发展主线，深化实施“依港立市、工业强市、开放活市、科教兴市”发展战略，突出项目、品牌、创新和服务带动，着力扩大内需拉动增长，优化结构提升质量，强化三农巩固基础，改善民生促进和谐，发挥优势冷静应对，坚定信心迎难而上，确保全市经济社会又好又快发展，以优异成绩迎接新中国成立六十周年。

　　经济社会发展的主要预期目标是：地区生产总值增长11%；财政总收入增长13%，其中地方级财政收入增长13.2%；规模工业产值增长16.6%；农业总产值增长4%；全社会固定资产投资增长20%；外贸出口增长10%；实际利用外资与上年持平；社会消费品零售总额增长15%，居民消费价格总水平涨幅控制在4%左右；城镇登记失业率控制在4.5%以内；人口自然增长率控制在7％。以内；城镇居民人均可支配收入实际增长7%,农民人均纯收入实际增长6%;单位生产总值能耗降低2.6%，二氧化硫排放量控制在2.0万吨以内，化学需氧量排放减少0.4%。在实际执行中力求更好更快些。

　　实现上述目标任务，必须着力做好以下六个方面工作：

　　（一）着力扩大内需，增强经济内生发展新动力。认真贯彻落实中央、省有关扩大内需促进增长的政策措施，千方百计抓项目、促投资、扩消费、拓市场，努力增强发展的内生动力。

　　把增加投资作为扩大内需的主要手段。一是千方百计加大政府投资。发挥政府投资导向作用与倍增效应，加大对农业农村、民生工程、基础设施、生态环境等方面的投入，引导全社会增加投资。投入60亿元以上建设交通基础设施、25亿元以上改善市政基础设施、15亿元以上加快发展社会事业、40亿元以上完善农业基础设施、15亿元以上改善生态环境。加强项目前期工作，主动向上沟通衔接，力争有更多项目进入国家、省扩大内需盘子。实行政府投资项目代建制，加强项目建设监管，确保项目建设质量。二是千方百计保障重点项目。今年安排市级在建重点项目195个，总投资1333.7亿元，年度投资257.9亿元，其中省级重点项目56个，总投资846.6亿元，年度投资163.7亿元。强化重点项目服务，及时解决项目建设中存在的问题，为项目建设提供有力要素保障。三是千方百计激活民间投资。认真落实支持民营经济发展的各项政策，加大政策宣传力度，定期发布产业政策导向和项目指引，举办民间投资专场推介会，推出一批面向社会资金、盈利预期良好的项目，引导民间资本积极进入。

　　把促进消费作为扩大内需的关键所在。一是增强居民消费能力。落实好规范公务员津贴制度、事业单位人员收入分配制度改革，扩大社会保障覆盖范围，提高社会保障标准和离退休人员待遇，积极发展消费信贷，增强居民消费购买力。二是培育居民消费热点。推行带薪休假，刺激节日消费，支持居民购房，扩大汽车、通信、旅游、休闲、教育、文化、健身等热点消费，多领域、多层次开发消费产品，努力满足不同层次群体的消费需求。三是开拓农村消费市场。认真组织家电、汽车下乡和商品供应工作，积极推进农村集贸市场升级改造和农家店建设，建成农资配送中心12个，发展连锁经营网点800个，大力拓展农村消费市场。四是改善居民消费环境。健全价格监测、预警和应急机制，加强价格监管，努力保持物价基本稳定。严格落实产品质量安全责任制，强化食品药品质量安全监管，加大对假冒伪劣产品和市场欺诈行为的整治力度，推进诚信体系建设，净化市场环境，促进安全消费。

　　把拓展市场作为扩大内需的有效措施。鼓励和组织企业外出参展促销，设立支持企业拓展市场专项资金，对企业在市外参展按省内每个展位3000元、省外每个展位5000元给予补助。引导企业参加福建营销联盟，组织名优产品到大中城市展销。鼓励企业与国内知名网站开展电子商务合作，建立漳州工业品专区。鼓励政府采购与集团采购优先采用本地名优产品。发挥龙头企业营销网络优势，引导中小企业融入龙头企业营销网络，整合行业力量共同开拓国内市场。

　　（二）着力强农惠农，实现城乡统筹发展新突破。认真贯彻落实党的十七届三中全会精神，按照省委提出“四重三农”的要求，有效促进农业增效、农民增收、农村繁荣，推进城乡一体化发展，推动新农村建设走在全省前列。

　　深化农村制度改革。稳定和完善农村基本经营制度，赋予农民更加充分而有保障的土地承包经营权，保持现有土地承包关系并长久不变。建立健全土地承包经营权流转市场，按照依法自愿有偿原则，采取转包、出租、互换、转让、股份合作等形式，促进土地承包经营权有序流转，推动农业规模化发展。建立耕地保护共同责任机制，落实“先补后占”政策，严格耕地保护。深入推进农村产权制度改革，巩固扩大集体林权制度改革成果，深化拓展海域、内陆水域使用权和小型农田水利设施产权制度改革。深化农村金融改革，创新农村金融产品，争取在村镇银行设立上有新突破。拓展农业政策性保险范围，加大对农业主导产业、渔船、渔工政策性保险试点的补助力度。加强村级“六大员”队伍建设，完善下派党员干部工作机制，选派大学生进村工作，增强农村组织保障能力。

　　加快现代农业发展。按照高产、优质、高效、生态、安全的要求，加快转变农业发展方式，提高农业综合生产能力。稳定粮食种植面积，扶持发展种粮大户，落实粮食订单收购直补、良种补贴和农机补贴政策，完善农资综合补贴动态调整机制，严格执行粮食最低收购价政策，确保粮食安全。大力发展特色农业，重点壮大八大优势产业，加强闽荷花卉合作，建成闽荷花卉交流合作中心，做大做强花卉产业。实施“强龙带动”工程，支持龙头企业领办农民专业合作组织，建基地、创品牌、拓市场。推进农村信息化建设，加快农业“五新”示范推广，积极推行测土配方施肥，增强科技对农业发展的支撑力。抓好农业标准化示范区建设，推进农业标准化生产，大力发展绿色食品、有机食品、无公害农产品。加快农产品质量安全检验检测体系建设，健全农产品市场准入机制，提高农产品质量安全水平。发挥海峡两岸农业合作实验区的先行先试优势，加快漳浦台湾农民创业园、海峡两岸(东山)水产品加工集散基地建设，积极筹建海峡两岸花卉集散中心，推进漳台农业合作上新水平。

　　提升公共服务水平。一是加大农村基础设施投入。开展土地开发整理和农田基本建设，建设4万亩高标准农田、1.8万亩省级商品粮基地。实施水利建设“五个二”工程和农村饮水安全工程，推进海堤、水库除险加固和防洪排涝、水土保持、清淤清障工作，加强防汛非工程体系建设，做好抗旱工作，健全防灾减灾应急处置机制。继续实施农村路网工程，提高行政村通车率。加快8条主要河流及中小流域综合治理，推进市县第二饮用水源、古雷供水工程、东山供水工程和长泰枋洋水利工程等项目前期工作。加快推进海洋与渔业减灾防灾“百千万”工程建设，扎实推进一批渔港建设。二是加大农村环境整治投入。抓好第二批20个市级社会主义新农村示范村建设，拓展新一轮农村“家园清洁行动”和“家园整理行动”，支持39个扶贫开发重点村建设。加快推进农村病死畜禽无害化处理池建设，建成农村户用沼气池1.5万口（户）。实施国债安全饮水项目，解决农村安全饮水问题。三是加大农村社会事业投入。落实被征地农民社会保障、农民最低生活保障、新型农村合作医疗三条保障线，探索渔民转业转产的扶持办法和保障措施。推进城乡社会事业统筹规划、同步发展，使更多优质社会事业资源进入农村。

　　（三）着力转型升级，构筑现代产业发展新格局。坚持制造与创造相促进、工业化与信息化相融合，构筑先进制造业与现代服务业双轮驱动、相互促动的现代产业发展新格局。

　　突破重点区域，增强发展支撑。一是打造沿海经济繁荣带。优化生产力布局，推进重大工业项目向环境容量高、立地条件好的沿海区域布局，提高产业空间集中度。加快厦漳跨海大桥、厦成高速漳州段、招银疏港高速、滨海大通道、漳州绕城高速、港尾铁路、古雷港区4#、8#、9#泊位等一批重大基础设施筹划建设，推进古雷开发区、漳州开发区、龙海经济开发区、南太武滨海工业集中区等重点区域开发，增强沿海区域对大工业项目的承载能力。二是提升工业园区集聚度。统筹现有省级以上开发区发展，加大投入力度，完善基础设施，整合壮大提升产业园区。建立健全以市场运作为主的开发机制、以完善服务体系为重点的协调推进机制，积极吸纳各类资本投资建设产业园区。制定出台产业导向明确、利益分配合理的政策措施，推行“飞地工业”发展模式，形成企业集群、产业集群和区域集群效应。三是推进县域发展新跨越。完善支持县域经济发展的各项政策措施，建立更加有效激励机制，推进各县域竞相发展。引导各县突出特色、找准重点，加快发展具有比较优势的特色产业，切实增强县域经济发展内在活力。加强县城建设与管理，完善配套设施和服务功能，提升县城的品位和形象，增强县城聚集带动能力。继续支持各县依托产业基础和文化特色举办各种节会，以节会促进氛围营造、项目引进、发展提速。

　　强化内涵发展，提升产业层级。一是强化项目带动，壮大产业规模。围绕“3+5”产业，努力培育百亿元级、千亿元级支柱产业。举全市之力，推进古雷石化、福欣特殊钢等重大产业项目建设，促其早建成、早投产。继续实施“千百工程”，积极推进旗滨玻璃、艺科环境科技、利胜电光源、龙翔实业、新福达汽车、创大太阳能、西冷光电、华福造船、毅宏游艇、日产游艇、三宝高强度钢、福钢运动器材、台湾玻璃、安安超细纤维等一批投资额在5亿元以上的重大工业项目建设，确保工业投资达180亿元以上。二是强化品牌带动，支撑产业扩张。开展“办名厂、创名牌、育名家”创建活动，引导企业做精做强主业，做优做响品牌，奋力进军产品、市场和价值链高端。引导品牌企业拉长品牌产品产业链，以发展品牌经济带动资源、产品和市场的整合。引进国际性、国家级品牌企业，借助外力壮大品牌群体。三是强化创新带动，提升产业质态。充分发挥企业创新主体作用，鼓励企业加大研发投入，设立研发平台，引进研发人才，提升创造能力。加强知识产权保护，支持建立国家钟表产品、体育用品质量检验中心，推进龙溪轴承重型车用关节轴承技术改造、福建力佳设备研发等一批研发项目建设，新建市级以上企业技术中心8个。积极推进四大产业化基地建设，加快发展新兴高新技术产业群体。引导企业抓住增值税转型改革的契机，加大技改投入，提高产业技术构成水平。完善产学研合作的模式与机制，引导企业与高等院校、科研院所共建创新载体，联合科技攻关。抓住全球金融危机引发人才回归潮的机遇，引进一批高层次人才。加大财政资金对科技创新的扶持力度，健全科技创新投融资机制，探索建立科技创新风险基金。四是强化服务带动，保障产业发展。落实各项优惠政策措施，用好省市各种发展基金，尽力帮助企业渡过难关。引导金融机构扩大信贷规模，建立龙头企业资金链保障机制，扶持信用担保机构发展，鼓励企业上市融资，探索企业集合发债，增强企业资金保障。积极对接国家产业振兴规划，及时出台扶持政策。深入开展“破难题、促发展”专项行动，实行“一企一策”，实实在在帮助企业化解难题。

　　壮大第三产业，促进互动发展。一是搭建制造业服务平台。优先发展与制造业相配套的生产性服务业，推进科技信息、工业设计、金融保险、会计服务、市场策划、法律咨询等新兴服务业加快发展，提升制造业发展配套服务水平。依托我市特有的区位优势和发达的港路运资源优势，大力发展总部经济、流量经济、道口经济，加快建设一批现代物流项目。二是构筑中心城区新商圈。组织实施城区商业网点规划，推进商业布局调整和资源整合。完善南昌路、新华片区核心商业区的整体功能，培植商贸精品新亮点。加快推进龙文明发商业广场等商贸项目建设，形成城东新商圈。配套完善城西商贸、文体等服务设施，构建城西新商圈。培育发展专业街区、楼宇经济，促进商贸服务业转型升级。加大区域性专业市场建设力度，全力打造龙门蔬菜批发市场、漳州花卉交易中心等一批专业市场。三是推进旅游目的地建设。支持南靖创建优秀旅游县，支持东山风动石、天福茶博物院创建国家5A级旅游区，南靖、华安土楼创建国家4A级旅游区，加快云霄翠丰海水温泉项目建设，加快创建五星级饭店步伐，推动滨海火山地质公园二期工程建设，推进灵通山申报国家地质公园。整合提升旅游资源，培育旅游精品线路，促进乡村旅游发展。四是保持房地产业健康发展。从强化金融支持、降低交易成本、完善住房公积金制度、改善供应体系等方面加大扶持力度，确保房地产业持续健康发展。落实鼓励房地产市场发展的各项政策，加强政策宣传，完善信息发布，引导合理消费，激活房地产一级市场，搞活房地产二级市场。

　　（四）着力改革开放，增创经济社会发展新优势。以纪念改革开放30周年为新起点，推进改革开放向更宽领域、更深层次发展，以深化改革激发活力，以扩大开放集聚要素，增创内力迸发、外力涌入、合力倍增的发展新优势。

　　提升漳台合作水平。认真贯彻胡锦涛总书记在纪念《告台湾同胞书》发表30周年座谈会上的重要讲话精神，持续拓展“五缘六求”优势，积极抢占“大三通”先机。加强港口软硬件建设，提升漳州港直通能力，加快八大物流园区建设，全力打造两岸物流中转基地。继续办好漳台经贸恳谈会、海峡两岸花博会等对台重大经贸活动，组织更多团组赴台招商，力促漳台产业深度对接。全力推进东山经济技术开发区扩区和南太武滨海工业集中区申报国家级台商投资区工作，高起点建设一批台商投资创业载体，吸引更多台资项目落地投产。发挥行业协会等民间组织的作用，加强与台湾工商社团、行业中介组织的联系，吸引台湾产业组团式转移。采取更加灵活有效措施，拓展对台小额贸易。加快建设台商综合服务大楼，支持天福茶学院发展和长泰创办台商子弟学校、智远职业技术学院。全方位、多层次拓展漳台祖地文化交流，进一步加强族谱对接，做好对台祖地宫庙等民间信仰交流工作，强化旅游对接往来，提升漳台合作水平。

　　增强招商引资实效。一是选准招商重点。紧紧围绕“引进一批龙头项目、培育一批特色板块、打造一批优势产业”的目标，紧盯世界500强、台湾100强、中央直属企业和民营大集团，加强沟通对接，跟踪投资意向，努力在招引重特大项目上取得新突破。二是拓展招商领域。既突出港台、主攻日韩、拓展欧美，巩固传统外资来源地，又瞄准长三角、珠三角、泉厦等民资活跃地区，开展密集式招商，争取从更大范围、更高层次上吸纳国内外生产要素流入我市。三是改进招商策略。充分利用我市良好的港口、交通、环保、区位等综合优势，释放扩张开发区、集中区的集聚效应，以开展招商竞赛为抓手，以办好节会展会为平台，坚持走专业化、市场化、集约化招商引资新路子，有针对性地采用敲门招商、定向招商、中介招商、代理招商、以商引商等多种方式，进一步增强招商引资实效。

　　力保出口持续增长。整合扶持外贸发展资金，支持企业扩大外贸出口，鼓励企业开展出口信用保险。认真贯彻落实中央出口退税政策，提高退税效率，及时拨付退税资金。加强我市出口商品的宣传推介，推动出口市场多元化。加大对东欧、中东、拉美、非洲等新兴市场的开拓力度，积极组织出口企业参加国（境）外的各类商品展销会。继续加强电子商务平台建设，推动企业运用现代信息手段开拓国际市场。

　　深化各项配套改革。推进国有企业改革与发展，建立健全现代企业制度，落实国有资产出资人和国有产权代表制度，实施国有资本收益收缴和经营预算制度。深化财税金融体制改革，实行积极财政政策，落实增值税转型改革，做好成品油税费改革相关衔接工作，推进金融产品创新。稳步实施政府机构改革，加快乡镇机构改革，深化行政审批制度改革，提升行政服务中心规范化运作水平。注重社会领域改革，加快事业单位分类改革。推进资源价格改革并形成机制，落实污水、垃圾处理和排污收费政策，促进公共资源市场化配置。

　　（五）着力生态建设，迈出宜居城乡建设新步伐。围绕建设生态名市目标，加强城乡规划建设管理，改善城乡生态环境，全力打造宜居城乡，让群众在良好的生态环境中生产生活。

　　以提升品位为重点，建设优美城乡环境。一是强化规划引领。深化中心城市拓展概念规划，加强城市设计，开展城市整体风貌特色规划，完善单体设计、街景立面、建筑风格、城市色彩、园林绿化等各项专项规划。统筹整合城乡资源，推进中心城区与次城区、主要县城之间在规划上实现统筹协调、功能互补。统筹城乡规划，加强镇、村规划编制，加快推进海西城镇群建设。二是完善城市功能。坚持市场化运作，增加城建投入，市区城建项目计划投入15亿元以上，加快城市路网建设，开工建设十四路二桥，迅速拉开城市框架。坚持资源共享、设施共用，进一步完善各项功能设施，重点抓好城区供水工程、燃气工程、通信网络工程、园林绿化工程等建设，加大市区内河整治力度，全面增强城市承载能力。完善交通管理，扶持公交发展，加快公交化进程。三是创新城市管理。进一步理顺城市管理体制，规范小区物业管理，加大城市管理行政执法力度，推进城市管理运行机制、管理方式的创新，实现城市管理的高效化、精细化和常态化，全力营造整洁美观有序的城市环境。

　　以节能减排为手段，营造优质人居环境。深入实施《漳州市循环经济发展规划纲要》，重点抓好68家重点能耗企业的节能降耗工作，大力发展循环经济，积极推行清洁生产，扶持发展再生资源回收利用产业，新增清洁生产试点企业5家。严格实施节地节能节水节材行业标准，引导和鼓励企业应用节能新技术、新工艺、新材料、新装备，淘汰落后生产能力。确保7个县和5个工业区的污水处理厂年内建成投入运行，加快中心市区、各县（市）和漳州、蓝田、金峰开发区污水配套管网建设。加快推进减排工程建设，突出以再生纸、食品等行业为重点，推进9家再生纸厂化学需氧量生化设施改造，提高废水综合利用率；加快5 家重点企业脱硫设施建设，加快漳州天然气(LNG)工程建设，提高工业和生活清洁能源使用率。

　　以生态保护为基石，优化和谐生态环境。严格落实环保责任制，推行环保问责制。严格实施环保规划，全面开展规划环境影响评价。加快生态名市建设，大力鼓励植树造林，推进绿色通道建设，加强自然保护区建设，加大生态林保护力度。抓紧编制《漳州市海洋环境保护规划》，提高海洋生态环境建设和海洋环境功能区保护水平。实施“碧海银滩”行动，开展重点滨海旅游区、重点养殖区、重点渔港和临港工业集中区的污染整治。加快垃圾处理设施建设，确保11个县（市、区）垃圾处理场全部建成投入使用。加强水环境综合整治，严格落实《强化九龙江流域水环境综合整治实施意见》，深入开展九龙江流域整治专项行动，严厉打击违法排污行为，确保流域水质达标。加强饮用水源保护，强化综合整治，强化设施建设，强化取水口巡查管理和水厂水质监测，健全饮用水安全应急预案，确保群众喝上干净安全的水。

　　以创建活动为载体，提升环境建设水平。巩固提高国家卫生城市、国家园林城市创建成果，开展“宜居社区”、“宜居城镇”、“宜居村庄”创建活动。推进新农村建设示范工作，继续开展“创绿色家园、建富裕新村”和“环境优美乡镇”、“生态村”创建活动，深入实施以“家园清洁行动”、“家园整理行动”为载体、以“三清六改”为主要内容的村容村貌整治工程，突出抓好农村垃圾清运、禽畜养殖业污染治理、沼气推广、旱厕填埋改造、农村卫生厕所建设，努力建设农村美好家园。

　　（六）着力民生民心，开创和谐漳州建设新局面。始终把实现人民群众最关心、最直接、最现实的利益作为各项工作的出发点和落脚点，继续实施一批为民办实事项目，切实增进“五有”建设实效，不断改善民生、凝聚民心。

　　完善社会保障体系。全面落实就业再就业政策，加强高校毕业生就业指导，努力解决下岗职工、返乡农民工再就业问题，加大就业困难人员、城镇零就业家庭的就业援助力度，支持扶助自主创业，完善职业技能培训制度，加大劳务派遣力度，推进农村富余劳动力转移，千方百计扩大城乡就业，确保新增就业5.8万人。扩大企业基本养老保险覆盖面，推进机关事业单位养老保险制度改革，完善被征地农民就业培训和养老保险制度，探索和建立新型农村养老保险制度，提升农村新型合作医疗管理水平，增加城镇居民医疗参保人数，做好政策性农业保险扩面工作，加快建立覆盖城乡的社会保障体系。继续增加对城市和农村低保补助，适当提高企业退休人员基本养老金水平。筹建市级社会福利中心和一批县级社会福利中心，新建10所乡镇敬老院，改扩建5所乡镇敬老院，再建一批农村幸福园。加快推进“金保工程”建设，提高社会保障服务水平。

　　加快发展社会事业。加强中小学教师队伍建设，大力实施素质教育，推进义务教育均衡发展，完善教育经费保障机制。实施农村寄宿制学校建设、校舍维修和抗震加固工程，推进农村中小学校布局结构调整，加强特教学校、幼儿园建设，积极推行幼儿早期教育。加强市区中小学改造提升，完成漳州实小新校区建设。大力发展职业教育，加强中职教育基础能力建设，推进县级职教中心建设。支持漳州师院、职业技术学院发展，支持卫生职业学院、城市职业学院人才培养评估工作和南靖职教中心创建国家级示范校。加快建立覆盖城乡的公共卫生服务体系，提升社区和农村卫生服务水平。新建市医院医技综合大楼、市医院朝阳分院门诊大楼、龙海第一医院门诊大楼，改扩建市中医院病房大楼，筹建市妇幼保健院、市精神卫生防治中心。改造提升20所乡镇卫生院，加强16所社区卫生服务中心基础设施建设、设备添置和人员技术培训。鼓励社会力量兴办民营医院，推进龙文医院、正兴医院加快建设。抓好重大传染病防治，提高重大疾病防控和突发公共卫生事件处置能力。稳定低生育水平，提高出生人口素质。积极备战第十四届省运会，办好第十一届市运会，动工兴建漳州体育场等一批城乡体育设施，实施全民健身工程。同时，做好第二次全国经济普查工作，抓紧第二轮修志，发展残疾人事业，加强妇女儿童、老干老龄、红十字会、慈善事业、民族宗教、外事侨务、审计、统计、物价、档案、气象、防震等工作。

　　持续改善人民生活。高度重视食品药品质量安全，健全责任体系，深化专项整治，加强监测监管，确保群众饮食用药安全。落实调高优抚对象和建国前老党员抚恤和生活补助标准，提高抗日战争时期参加革命的离休干部护理费标准，提高特殊对象补助水平。加大保障性住房建设力度，解决全市2500户城市低收入家庭住房困难问题，其中实物配租1500户、发放租赁补贴1000户。实施“造福工程”和扶贫开发“整村推进”，支持原中央苏区县、老区、少数民族地区、边远地区加快发展，加强山海协作和对口帮扶工作，确保援川救灾工作三年任务两年完成，做好库区移民后扶工作。

　　推进文化发展繁荣。加强现代公民素质教育，深入开展“爱我漳州，从我做起”活动，认真做好道德标准评选工作，培育爱岗敬业、创业创新、诚实守信精神。加快市区街道文化站和乡镇文化站建设，积极拓展文化“六进农家”的广度和深度，大力实施“农家书屋”等文化惠民工程，充分发挥农村宣传文化中心户“百千万工程”的作用，壮大村文化协管员和文化辅导员队伍。筹建市新图书馆、文化艺术中心、高山族博物馆，扩建红军进漳纪念馆，加快闽南文化生态保护区建设，加强非物质文化遗产保护与挖掘，推进各县（市、区）建设一批有特色的闽南文化展示点。完成第三次文物普查第二阶段工作，抢救维护文物，加强涉台文物保护。实施《文化强市纲要》，深化文化体制改革，鼓励引导民间资本投资发展文化产业，推进国际艺术交流中心、闽台文化影视城、海峡文化交流基地建设，推动文化产业、创意产业新发展。

　　全力维护社会稳定。强化信访工作责任制，加强社会舆情汇集分析，健全多元化矛盾纠纷解决机制，加大信访督办力度，积极预防和妥善处置群体性事件。全面贯彻党和国家的民族政策，依法管理宗教事务。深入开展平安漳州创建活动，加强社会治安综合治理，依法严厉打击各种刑事犯罪。强化“安全发展”理念，严格落实“一岗双责”和行政问责，全面启动企业安全生产主体责任“三年行动”，深入开展安全生产专项整治和隐患排查治理，坚决遏制重大安全事故发生。加强地质灾害治理、海上救助体系建设等工作，完善各类应急预案，增强应急处置能力。做好国家安全、国防动员、人民防空、支前双拥、打击制售假烟等工作。

　　三、以改革创新精神推进政府自身建设

　　面对国内外经济形势复杂多变的严峻挑战，面对“海西建设、漳州先行”的庄严使命，各级政府要主动适应形势变化，跟上时代发展步伐，切实加强政府自身能力建设，以能力大提升推进工作大创新，以工作大创新促进作为大拓展，努力开创政府工作新局面。

　　把握大势，科学决策。越是困难，越要做到科学决策，增强工作的前瞻性与有效性。切实学好政策、用活政策，加强对形势变化的研判分析和政策调整的跟踪衔接，把握好政策取向，谋划好工作思路，制定好应对措施。完善行政决策的程序和机制，健全重大行为决策听证、论证、合法性审查、集体决策制度，确保决策科学合理、施政有力有效。

　　创新运作，破解难题。在前所未有的困难面前，要以前所未有的工作责任心、前所未有的工作力度、前所未有的工作作风，大胆创新运作办法，全力推动难题破解。坚持“干”字当先，从最迫切的问题入手，从最薄弱的工作抓起，从最关键的环节突破，靠实干体现作为，靠实干拓展成效，靠实干推动发展。坚持“快”字为要，快速对接上级政策，快速落实工作部署，快速推进项目建设，以最快速度将每个利好政策转变为发展举措，尽最大努力把每个发展机遇转变为发展实效。

　　依法行政，依规办事。深入贯彻监督法及实施办法，认真执行市人大及其常委会的决议决定，自觉接受市人大及其常委会的法律监督、工作监督和市政协的民主监督。认真落实国务院《关于加强市县政府依法行政的决定》，大力推进法治政府建设，严格规范行政自由裁量权，加强和改善行政执法，提高依法行政水平。深入实施政府信息公开条例，完善政府门户网站，拓展政务公开范围，推进政府与公民的互动，建设规范透明的政务环境。

　　从严治政，清正廉洁。认真贯彻落实中纪委十七届三次全会精神，严格执行党风廉政建设各项规定，深入推进政府系统反腐倡廉建设。完善惩治和预防腐败体系，拓展从源头上预防和治理腐败领域，严厉查处权力滥用、商业贿赂、以权谋私等违法违纪案件，坚决纠正损害群众利益的不正之风。加强政府专门监督，强化行政效能监察，加大工作督查力度，进一步提升政府的执行力和公信力。

　　各位代表，海西建设势如虹，龙江潮声催先行。让我们更加紧密地团结在以胡锦涛同志为总书记的党中央周围，高举中国特色社会主义伟大旗帜，在省委、省政府和市委的正确领导下，紧紧依靠全市人民，坚定信心、有效作为，攻坚克难、奋勇争先，以优异的成绩迎接新中国成立六十周年，为推动漳州更好更快发展、开创漳州更加美好的未来而努力奋斗
